# Triglyceride lipase PNPLA2–independent suppression of c-MYC signaling by the metabolic coactivator ABHD5 in prostate cancer

**DOI:** 10.1016/j.jbc.2025.111001

**Published:** 2025-12-03

**Authors:** Aaron Lotvola, Guohua Chen, Guoli Zhou, James G. Granneman, Jian Wang

**Affiliations:** 1 Department of Oncology, Wayne State University School of Medicine, Detroit, Michigan, USA; 2 Department of Pathology, Wayne State University School of Medicine, Detroit, Michigan, USA; 3 Center for Statistical Training & Consulting, Michigan State University, East Lansing, Michigan, USA; 4 Center for Molecular Medicine and Genetics, Wayne State University School of Medicine, Detroit, Michigan, USA

**Keywords:** ABHD5, c-MYC, PNPLA2, lipid metabolism, tumor suppressor

## Abstract

The *MYC* oncogene encodes a transcription factor that regulates cell growth, metabolism, and proliferation. Its dysregulation is a hallmark of many cancers, including prostate cancer. Elevated c-MYC expression promotes tumor progression and therapy resistance, yet c-MYC remains a challenging therapeutic target because of its intrinsically disordered structure and lack of enzymatic activity. Identifying upstream regulators of MYC activity may reveal new therapeutic strategies. α/β-Hydrolase domain–containing protein 5 (ABHD5) is best known as a coactivator of the triglyceride lipase PNPLA2, facilitating intracellular lipolysis. However, recent studies have suggested a tumor-suppressive role for ABHD5 in various cancers, including prostate cancer, though the molecular mechanisms remain unclear. Here, we identify ABHD5 as a suppressor of c-MYC-driven transcriptional programs in prostate cancer cells. Transcriptomic profiling in 22Rv1 cells revealed that ABHD5 overexpression downregulates MYC target genes and reduces c-MYC protein levels. In contrast, ABHD5 knockout increased c-MYC protein expression, enhanced cell proliferation, and markedly elevated colony-forming capacity. ABHD5 deficiency also conferred resistance to the pharmacological c-MYC inhibitor 10058-F4. Notably, PNPLA2 knockout failed to phenocopy these effects, indicating that the tumor-suppressive function of ABHD5 is independent of its canonical lipolytic role. Furthermore, ABHD5 overexpression continued to suppress c-MYC in PNPLA2-deficient cells, confirming a lipase-independent mechanism. These findings define a previously unrecognized role for ABHD5 as a negative regulator of c-MYC and highlight a novel, noncanonical pathway linking lipid metabolism regulators to oncogene control in prostate cancer.

The *MYC* oncogene encodes a pleiotropic transcription factor that plays a central role in regulating cell growth, metabolism, and proliferation (1). Dysregulation of c-MYC is a hallmark of many human cancers, where its overexpression drives malignant transformation and tumor progression by activating a broad transcriptional network controlling ribosome biogenesis, nucleotide synthesis, and metabolic reprogramming (2). In prostate cancer, elevated c-MYC expression is frequently observed and strongly correlates with disease aggressiveness, therapy resistance, and poor clinical outcomes (3, 4, 5, 6). Despite its well-established role in tumorigenesis, c-MYC remains a challenging therapeutic target because of its intrinsically disordered structure, lack of defined ligand-binding pockets, and extensive involvement in essential cellular functions (7). These limitations underscore the need to identify alternative mechanisms that regulate c-MYC expression and activity in cancer cells, which may reveal novel therapeutic vulnerabilities.

Recent studies have identified the lipolytic coactivator α/β-hydrolase domain–containing protein 5 (ABHD5) as a critical regulator of intracellular lipid metabolism and a potential tumor suppressor in several malignancies. ABHD5 functions as a coactivator of the triglyceride lipase PNPLA2 (also known as adipose triglyceride lipase), promoting the hydrolysis of stored triglycerides within lipid droplets to generate free fatty acids (8, 9). Beyond its canonical role in lipid catabolism, emerging evidence suggests that ABHD5 contributes to tumor suppression by regulating lipid mobilization, energy stress signaling, and cellular metabolic homeostasis (10, 11). In prostate cancer, ABHD5 expression is often reduced, and its loss is associated with enhanced lipid accumulation and tumorigenicity (12, 13). Although PNPLA2 has been recognized as a downstream effector of ABHD5, it remains unclear whether ABHD5 exerts its tumor-suppressive functions exclusively through PNPLA2-mediated lipolysis. Clarifying both PNPLA2-dependent and -independent roles of ABHD5 in prostate cancer may uncover new insights into how lipid metabolism intersects with oncogenic signaling pathways such as c-MYC.

In this study, we identified ABHD5 as a novel suppressor of c-MYC-driven transcriptional programs in prostate cancer cells through unbiased transcriptomic profiling. Using systematic gain- and loss-of-function approaches, we demonstrated that ABHD5 represses c-MYC expression, inhibits cell proliferation, and enhances sensitivity to c-MYC inhibition—independently of its canonical lipolytic effector PNPLA2. These findings reveal a noncanonical, lipase-independent role for ABHD5 in restraining oncogenic c-MYC signaling and highlight its potential as a therapeutic target in MYC-driven prostate cancer.

## Results

### ABHD5 suppresses c-MYC-driven transcriptional programs in prostate cancer cells

**Figure 1:**
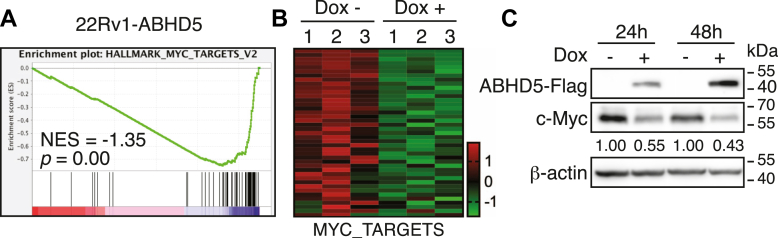
**ABHD5 suppresses c-MYC signaling in prostate cancer cells.***A*, GSEA of RNA-Seq data from 22Rv1 cells with doxycycline (Dox)-inducible ABHD5 identified significant downregulation of the HALLMARK_MYC_TARGETS_V2 pathway. Cells were treated with Dox for 48 h. *B*, heatmap showing repression of 36 MYC target genes upon ABHD5 induction. *C*, Western blot analysis confirmed a time-dependent reduction in c-MYC protein following ABHD5 expression. β-actin served as a loading control. ABHD5, α/β-hydrolase domain–containing protein 5; GSEA, gene set enrichment analysis.

We previously showed that ABHD5 exerts strong antiproliferative effects in prostate cancer cells (12, 13). To elucidate the underlying molecular mechanism, we performed transcriptomic analysis in 22Rv1 prostate cancer cells expressing a doxycycline (Dox)-inducible ABHD5-FLAG construct (12). Gene set enrichment analysis (GSEA) (14) of RNA-Seq data revealed that the HALLMARK_MYC_TARGETS_V2 gene set was among the most significantly downregulated pathways following ABHD5 induction (Fig. 1*A*). Notably, 36 of 70 genes in the MYC target gene set were significantly repressed, and hierarchical clustering confirmed broad suppression of these targets in ABHD5-overexpressing cells (Fig. 1*B*). To examine whether these transcriptomic changes were associated with alterations in c-MYC protein levels, we performed Western blot analysis following Dox induction. ABHD5 expression led to a time-dependent reduction in c-MYC protein abundance at 24 and 48 h postinduction (Fig. 1*C*). These findings indicate that ABHD5 negatively regulates c-MYC-driven transcriptional programs by suppressing c-MYC protein expression and suggest a novel tumor-suppressive mechanism in prostate cancer cells.

### ABHD5 is required for suppression of c-MYC protein expression in prostate cancer cells

**Figure 2:**
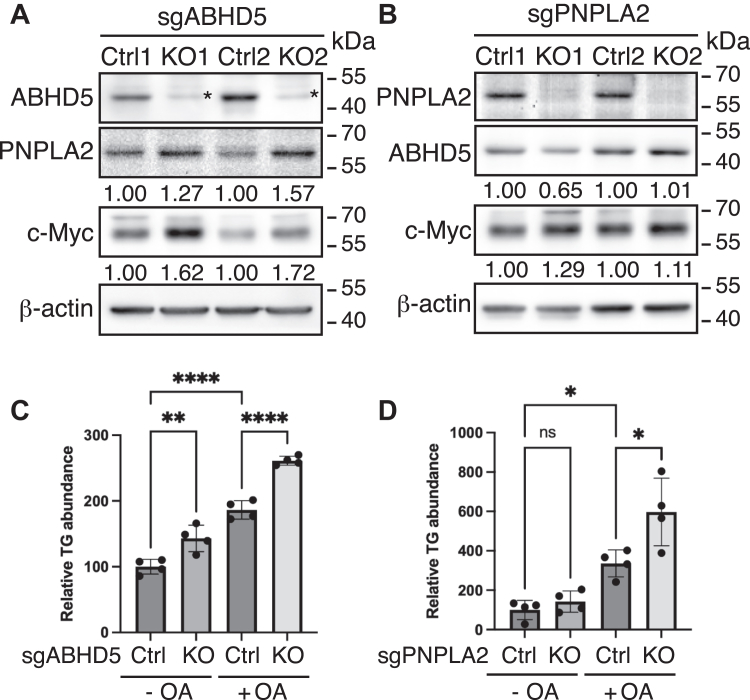
**ABHD5, but not PNPLA2, is required for suppression of c-MYC protein expression.***A* and *B*, Western blot validation of CRISPR–Cas9-mediated knockout of ABHD5 or PNPLA2 and evaluation of the effects on c-MYC protein in 22Rv1 cells. β-actin was used as a loading control. *C* and *D*, triglyceride assay showing elevated intracellular triglyceride accumulation in ABHD5- or PNPLA2-deficient cells following 14 h of oleic acid (OA) treatment. The relative abundance was calculated by normalizing each measurement to that of control cells without OA treatment. Data are presented as mean ± SD (independent biological replicates, ∗*p* < 0.05, ∗∗*p* < 0.01, ∗∗∗∗*p* < 0.0001, ANOVA test). ABHD5, α/β-hydrolase domain–containing protein 5.

To evaluate the functional consequence of ABHD5 loss on c-MYC protein expression, we generated ABHD5-deficient 22Rv1 cells using CRISPR–Cas9-mediated gene targeting. Guide RNAs targeting ABHD5 were stably transfected, and drug-resistant clones were selected with blasticidin. Western blotting confirmed successful ABHD5 knockout in selected clones, whereas clones retaining ABHD5 expression served as controls. In parallel, PNPLA2, the canonical lipolytic effector of ABHD5, was also targeted using single guide RNAs (sgRNAs). Two independent sgRNAs for each gene (sgABHD5-1/-2 and sgPNPLA2-1/-2) yielded robust and specific protein depletion as validated by immunoblotting (Fig. 2, *A* and *B*, top panels).

As expected, knockout of either ABHD5 or PNPLA2 resulted in substantial intracellular triglyceride accumulation upon oleic acid loading, confirming impaired lipolysis (Fig. 2, *C* and *D*). Interestingly, loss of ABHD5 led to a moderate upregulation of PNPLA2 protein, suggesting a potential compensatory response to lipolytic dysfunction. In contrast, PNPLA2 deletion had minimal effect on ABHD5 expression (Fig. 2, *A* and *B*, middle panels).

Strikingly, c-MYC protein levels were markedly elevated in ABHD5 knockout cells but remained unchanged in PNPLA2-deficient cells (Fig. 2, *A* and *B*, bottom panels). These findings suggest that while both ABHD5 and PNPLA2 are involved in lipid mobilization, only ABHD5 is required to suppress c-MYC protein expression, indicating a lipolysis-independent mechanism by which ABHD5 controls oncogenic MYC signaling in prostate cancer cells.

### Deletion of ABHD5 promotes cell proliferation and increases chemoresistance to c-MYC inhibition

**Figure 3:**
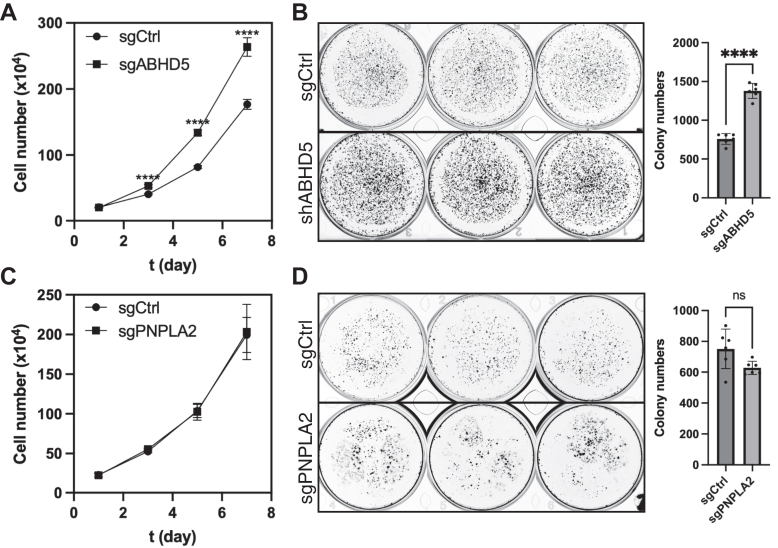
**ABHD5 deletion promotes prostate cancer cell proliferation and colony formation.***A*, growth kinetics showing increased proliferation in ABHD5-deficient 22Rv1 cells compared with controls (∗∗∗∗*p* < 0.0001, n = 4, *t* test). *B*, colony-formation assay showing enhanced colony numbers in ABHD5 knockout cells (independent biological replicates, ∗∗∗∗*p* < 0.0001, *t* test). *C* and *D*, PNPLA2 knockout had no effect on proliferation and modestly reduced colony formation (independent biological replicates, ∗∗∗*p* < 0.001, *t* test). Data represent mean ± SD. ABHD5, α/β-hydrolase domain–containing protein 5.

To determine the functional significance of ABHD5-mediated c-MYC suppression in prostate cancer cell growth, we examined the proliferative capacity of ABHD5-deficient 22Rv1 cells. Compared with control cells, ABHD5 knockout significantly enhanced cell proliferation, as shown by growth kinetics analysis (Fig. 3*A*). In a colony-formation assay, ABHD5 deletion nearly doubled the number of colonies, indicating increased tumorigenic potential (Fig. 3*B*). In contrast, deletion of PNPLA2 did not affect proliferation and colony formation (Fig. 3, *C* and *D*), further supporting a unique role for ABHD5 in suppressing cancer cell aggressiveness.

**Figure 4:**
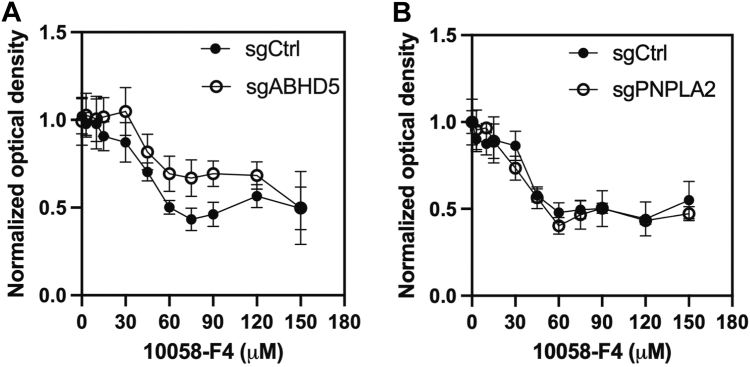
**ABHD5 deletion reduces sensitivity to c-MYC inhibition.***A*, cytotoxicity analysis showing that ABHD5-deficient 22Rv1 cells exhibited a higher EC_50_ for the c-MYC inhibitor 10058-F4 compared with control cells, indicating reduced drug sensitivity (∗*p* < 0.05, Wilcoxon rank sum test). *B*, PNPLA2 knockout had no effect on the cytotoxic response to 10058-F4. Data are presented as median ± interquartile range. ABHD5, α/β-hydrolase domain–containing protein 5.

To assess whether ABHD5-mediated regulation of c-MYC affects drug sensitivity, we treated cells with the c-MYC inhibitor 10058-F4 (15) and measured cytotoxic responses. Loss of ABHD5 significantly increased the EC_50_ of 10058-F4 (from 42.9 ± 12.3 μM to 55.4 ± 35.5 μM, *p* < 0.05), suggesting reduced sensitivity to MYC inhibition (Fig. 4*A*). In contrast, PNPLA2 knockout had no effect on drug response (Fig. 4*B*). These results highlight the functional importance of the ABHD5–c-MYC axis in regulating prostate cancer cell proliferation and responsiveness to MYC-targeted therapies.

### ABHD5 inhibits c-MYC expression independently of PNPLA2

**Figure 5:**
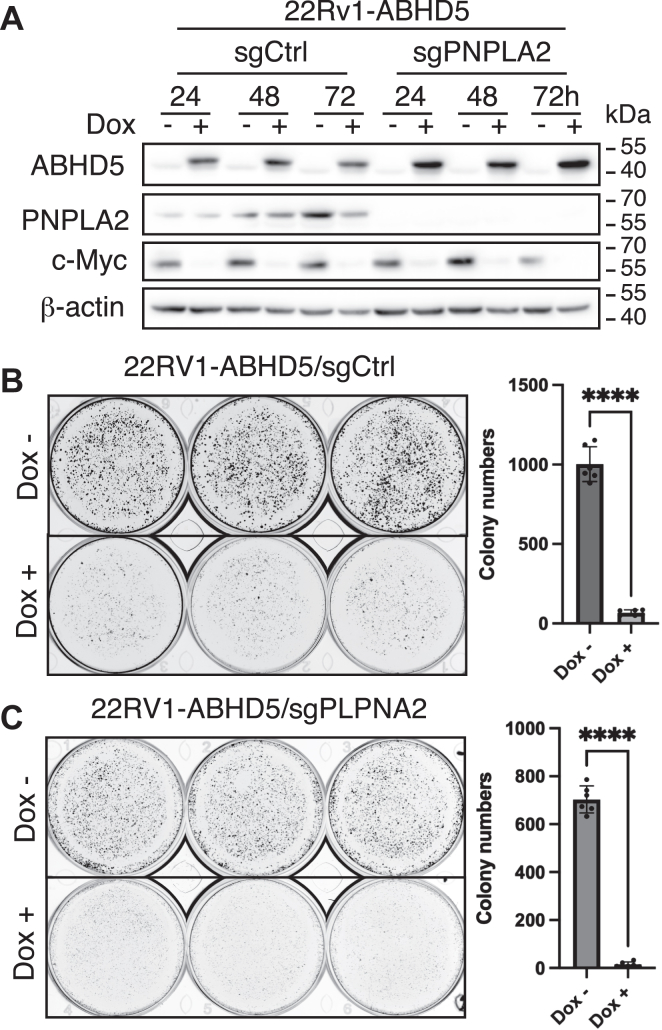
**ABHD5 suppresses c-MYC expression independently of PNPLA2.***A*, Western blot showing that ABHD5 overexpression reduced c-MYC protein levels in PNPLA2-deficient 22Rv1 cells. *B* and *C*, functional assays showing that ABHD5 induction suppressed proliferation (*B*) and colony formation (*C*) both in the presence or the absence of PNPLA2. Data are shown as mean ± SD (independent biological replicates, ∗∗∗∗*p* < 0.0001, *t* test). ABHD5, α/β-hydrolase domain–containing protein 5.

To determine whether PNPLA2-mediated lipolytic activity is necessary for ABHD5-dependent suppression of c-MYC, we generated a double-mutant 22Rv1 cell line lacking PNPLA2 in the background of Dox-inducible ABHD5 overexpression. Despite the absence of PNPLA2, ABHD5 overexpression retained its ability to downregulate c-MYC protein levels (Fig. 5*A*). Functionally, ABHD5 induction in PNPLA2-deficient cells still significantly reduced cell proliferation (Fig. 5*B*) and colony-forming capacity (Fig. 5*C*), comparable to effects observed in PNPLA2-intact cells. These findings demonstrate that ABHD5 suppresses c-MYC-driven oncogenic activity through a mechanism that is independent of PNPLA2-mediated lipolysis, suggesting a previously unrecognized noncanonical signaling role for ABHD5 in regulating MYC expression and cancer cell growth.

### Pharmacological activation of ABHD5 suppresses c-MYC expression in prostate cancer cells

**Figure 6:**
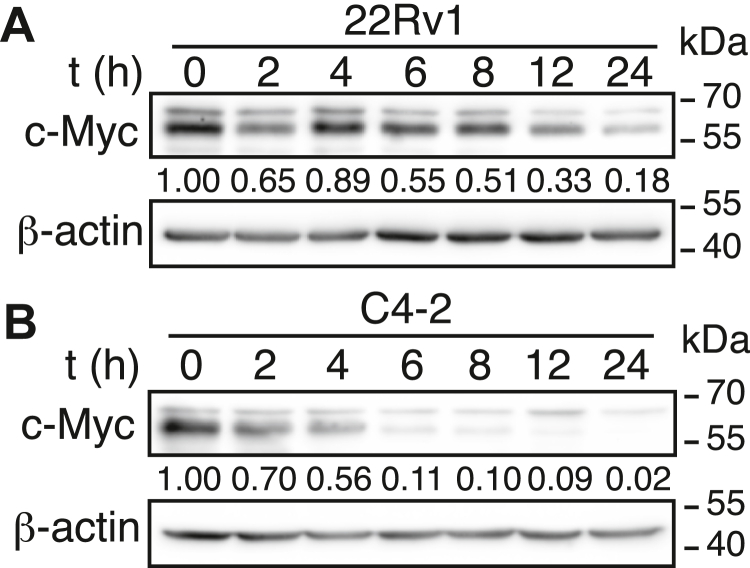
**Time-dependent suppression of c-MYC by ABHD5-activating ligand SR3420 in 22Rv1 and C4-2 cells.** 22Rv1 (*A*) and C4-2 (*B*) prostate cancer cells were treated with 10 μM SR3420, a prototype ABHD5-activating ligand, for the indicated durations. c-MYC protein levels were measured by Western blotting. β-Actin was used as a loading control. ABHD5, α/β-hydrolase domain–containing protein 5.

We treated 22Rv1 and C4-2 prostate cancer cells with SR3420, a synthetic ligand that activates ABHD5 by releasing it from its intracellular repressors (16). Western blot analysis revealed that SR3420 treatment led to a robust, time-dependent decrease in c-MYC protein levels in both cell lines (Fig. 6). These findings demonstrate that pharmacological activation of ABHD5 is sufficient to suppress c-MYC expression in prostate cancer cells.

## Discussion

This study identifies ABHD5 as a novel suppressor of c-MYC-driven oncogenic signaling in prostate cancer, revealing a previously unrecognized, lipase-independent mechanism underlying its tumor-suppressive activity. Although ABHD5 is classically characterized as a coactivator of the triglyceride lipase PNPLA2, our findings demonstrate that ABHD5 represses c-MYC transcriptional programs and protein expression independently of its canonical lipolytic function. These insights broaden the functional landscape of ABHD5 and establish a mechanistic connection between lipid metabolic regulators and oncogene control in prostate cancer.

c-MYC is a central driver of prostate tumorigenesis and is frequently overexpressed in both localized and advanced disease (17, 18, 19). Despite its clinical relevance, c-MYC has remained a challenging therapeutic target because of its intrinsically disordered structure, lack of druggable domains, and essential roles in normal cellular physiology. Prior attempts at direct pharmacological inhibition of c-MYC have achieved limited success (20, 21), underscoring the need to identify upstream regulators that modulate MYC expression and activity. In this study, we show that ABHD5 robustly suppresses MYC protein levels and target gene expression. Functionally, ABHD5 deficiency enhances proliferation, increases colony formation, and confers resistance to pharmacological MYC inhibition, highlighting the biological significance of this regulatory axis.

A key finding is that ABHD5 suppresses c-MYC through a mechanism independent of PNPLA2. Although knockout of either ABHD5 or PNPLA2 results in intracellular triglyceride accumulation, only ABHD5 deficiency promotes c-MYC upregulation and enhances oncogenic phenotypes. Moreover, ABHD5 retains its ability to suppress c-MYC expression and inhibits proliferation in PNPLA2-null cells, supporting a noncanonical, PNPLA2-independent role for ABHD5 in modulating oncogenic signaling.

We previously demonstrated that ABHD5 stimulates AMP-activated protein kinase and inhibits mTOR signaling (12). However, pharmacological inhibition of AMP-activated protein kinase fails to reverse ABHD5-mediated MYC protein suppression (data not shown), indicating that ABHD5 represses c-MYC independently of its role in energy stress and growth signaling. These observations suggest that ABHD5 has a distinct and specific capacity to inhibit c-MYC, contributing to its tumor-suppressive function.

The precise mechanism by which ABHD5 downregulates c-MYC remains to be elucidated. Several possibilities emerge from our data. ABHD5 may regulate upstream signaling pathways or activate transcriptional repressors that suppress MYC independently of its lipolytic role. Alternatively, ABHD5 may influence c-MYC through protein–protein interactions or organelle-specific signaling at the lipid droplet, mitochondria, or endoplasmic reticulum, thereby affecting c-MYC stability or transcriptional activity. Given the interaction of ABHD5 with other PNPLA family members, such as PNPLA1 (22) and PNPLA3 (23), it is also possible that ABHD5 modulates MYC expression *via* enzymes other than PNPLA2. For example, functional interaction between ABHD5 and PNPLA1 could affect c-MYC expression through ceramide-dependent signaling (24). Future studies aimed at defining the ABHD5 interactome, mapping its subcellular localization, and identifying downstream effectors will be crucial for mechanistic insight.

These findings also carry important therapeutic implications. We demonstrate that pharmacological activation of ABHD5 with the synthetic ligand SR-3420 robustly suppresses c-MYC protein levels in two castration-resistant prostate cancer models expressing either wild-type or constitutively active, alternatively spliced androgen receptor variants (25). Because c-MYC amplification drives the development of castration resistance (26, 27), targeting the ABHD5–c-MYC axis may offer an effective therapeutic approach for advanced prostate cancer. As a repressor of c-MYC, restoration or pharmacological activation of ABHD5 function may represent a promising strategy for targeting MYC-driven prostate tumors, particularly those with c-MYC overexpression and few actionable mutations. Notably, since ABHD5 suppresses *c-MYC* independently of its lipolytic activity, strategies that enhance ABHD5 function could minimize potential metabolic side effects associated with lipid breakdown. Thus, targeting metabolic regulators like ABHD5 that converge on oncogenic nodes such as c-MYC may provide a novel therapeutic avenue for prostate and other MYC-driven cancers.

In summary, our study uncovers a previously unrecognized function of ABHD5 in repressing c-MYC-driven oncogenesis through a PNPLA2-independent mechanism. These findings expand the known functions of ABHD5 beyond lipid metabolism and highlight its potential as a therapeutic regulator of oncogenic signaling in prostate cancer.

## Experimental procedures

### Cell culture, plasmid constructs, and cell line generation

Human prostate cancer 22Rv1 and C4-2 cells (American Type Culture Collection) were maintained in RPMI1640 medium supplemented with 10% fetal bovine serum, 100 U/ml penicillin, and 0.1 mg/ml streptomycin, and authenticated with short tandem repeat profiling. The pharmacological c-MYC inhibitor 10058-F4 was obtained from Thermo Scientific.

For CRISPR–Cas9-mediated gene targeting, sgRNA sequences against human *ABHD5* or *PNPLA2* were designed using an online bioinformatics tool (http://crispr.mit.edu). The oligonucleotides encoding sgRNAs were annealed and cloned into pSpCas9(BB)-2A-Bsd, a modified version of pSpCas9(BB)-2A-Puro (PX459; Addgene) in which the puromycin resistance cassette was replaced with a blasticidin resistance gene.

The sgRNA sequences used were as follows:

ABHD5: 5′-TTAAGGTGTGATATAGACGT-3′ and 5′-TTGGACGAAGTAGTAGACCC-3′

PNPLA2: 5′-CGGCGTCTACTACGTCGGCG-3′ and 5′-ACGTGGAACATCTCGTTCGC-3′

To generate knockout models, 22Rv1 or 22Rv1-ABHD5 cells were transfected with pSpCas9(BB)-2A-Bsd vectors carrying sgRNAs targeting *ABHD5* or *PNPLA2*, followed by selection with 5 μg/ml blasticidin. Drug-resistant clones were validated by Western blotting for loss of ABHD5 or PNPLA2 protein expression.

### RNA-Seq transcriptomic profiling and GSEA

Total RNA was isolated using TRIzol (Invitrogen), and complementary DNA libraries were prepared with the QuantSeq 3′ mRNA-Seq REV Library Prep Kit (Lexogen). Libraries were assessed on a TapeStation (Agilent) and sequenced (100-bp single end) on an Illumina HiSeq 2500 at the Wayne State University Applied Genomics Technology Center.

Raw sequencing reads (FASTQ) were processed with Trimmomatic (28) to remove low-quality and unassigned sequences. Cleaned reads were aligned to the human reference genome (hg19) using Bowtie2 (29), and transcript abundance was quantified in CPM with eXpress (30). Differential expression was analyzed with edgeR (31), and RNA-Seq data are deposited in Gene Expression Omnibus (accession no.: GSE151957).

Pathway enrichment was performed using GSEA (http://software.broadinstitute.org/gsea) with hallmark gene sets, 1000 permutations, preranked gene lists, and the classic enrichment statistic.

### Western blotting

Cells were washed twice with PBS and lysed in cytoplasmic lysis buffer containing 25 mM Tris–HCl (pH 7.5), 40 mM NaCl, 50 mM NaF, 2 mM Na_2_VO_4_, and 1% Triton X-100. Protein concentrations were determined using the BCA assay (Pierce). Equal amounts of protein (40 μg) were resolved by SDS-PAGE and transferred onto nitrocellulose membranes. Membranes were blocked with 5% nonfat dry milk and incubated with primary antibodies against ABHD5 (catalog no.: 12201-1-AP; Proteintech; 1:1000 dilution), PNPLA2/adipose triglyceride lipase (catalog no.: 2138; Cell Signaling; 1:1000 dilution), c-MYC (catalog no.: 10828-1-AP; Proteintech; 1:1000 dilution), FLAG M2 (F3165; Sigma; 1:5000 dilution), and β-actin (A2066; Sigma; 1:1000 dilution). After washing with PBS containing 0.1% Tween-20, membranes were incubated with horseradish peroxidase–conjugated goat anti-rabbit IgG or anti-mouse (Sigma; 1:5000 dilution). The ABHD5 and PNPLA2 antibodies were validated through CRISPR-mediated knockout in the current study, whereas the c-MYC antibody was validated by its vendor. Protein bands were visualized using enhanced chemiluminescence (PerkinElmer) on a C600 imaging system (Azure Biosystems). Densitometric quantification of immunoblots was performed using ImageJ (1.54g, NIH) software. Relative protein abundance was calculated by normalizing each target signal to its corresponding β-actin signal, followed by expressing the normalized values as a ratio relative to those of control cells.

### Measurement of intracellular triglycerides

Cells were cultured in the absence or the presence of 200 μM oleic acid, harvested, and suspended in 5% NP-40 aqueous solution. Cell extracts were prepared by two cycles of heating (95 °C, 5 min) and cooling (on ice, 5 min), followed by centrifugation at 12,000*g* for 5 min. Cellular triacylglycerol levels were quantified using a triglyceride reagent (catalog no.: 998-02992; Fujifilm) and measured on a microplate reader (BMG Labtech) according to the manufacturer’s instructions.

### Cell proliferation, colony-formation, and cytotoxicity assays

For growth kinetics, cells were seeded in 6-cm plates and cultured for the indicated time intervals in the presence or the absence of doxycycline (2 μg/ml). Dead cells were excluded by trypan blue staining (Invitrogen), and viable cells were counted using a hemocytometer under a light microscope.

For colony formation, single cells were seeded in 6-well plates and grown for 8 days. Colonies were fixed with 4% paraformaldehyde, stained with 0.4% crystal violet, and quantified using a GelCount system (Oxford Optronix).

Cells were seeded in 96-well plates and treated with increasing concentrations of the c-MYC inhibitor 10058-F4 for an additional 48 h. Cell viability was assessed using the CellTiter 96 AQ_ueous_ Cell Proliferation Assay (Promega). EC_50_ values were calculated using the drc R package (32). Group differences were evaluated using the Wilcoxon rank sum test (Mann–Whitney *U* test), with the null hypothesis that EC_50_ distributions were equal. Data are reported as median ± interquartile range.

### Statistical analysis

All data are presented as mean ± SD, unless otherwise indicated. Statistical analyses were performed using GraphPad Prism (version 10.6, Dotmatics) or R (version 4.40, R Core Team). For comparisons between two groups, unpaired two-tailed Student’s *t* tests were applied for normally distributed data. For comparisons involving more than two groups, one-way ANOVA was performed.

## Data availability

The RNA-Seq data analyzed in this study have been deposited in the Gene Expression Omnibus under accession number GSE151957. All other relevant data are available in the article.

## Supporting information

This article contains supporting information.

## Conflict of interest

The authors declare that they have no conflicts of interest with the contents of this article.
