# Supplementary material for: Triglyceride lipase PNPLA2–independent suppression of c-MYC signaling by the metabolic coactivator ABHD5 in prostate cancer
Source: J Biol Chem. 2025 Dec 3;302(1):111001. doi: 10.1016/j.jbc.2025.111001 (PMC12800686; doi:10.1016/j.jbc.2025.111001)
Supplement: Supporting information [file mmc1.pdf]

Triglyceride lipase PNPLA2-independent suppression of c-MYC signaling by the metabolic coactivator ABHD5 in prostate cancer

Aaron Lotvola[1], Guohua Chen[2], Guoli Zhou[3], James G. Granneman[4] and Jian Wang[2*]

Department of [1]Oncology, [2]Pathology, and [4]Center for Molecular Medicine and Genetics, Wayne State University School of Medicine, Detroit, MI 48202, USA

[3]Center for Statistical Training & Consulting, Michigan State University, East Lansing, MI 48824 USA

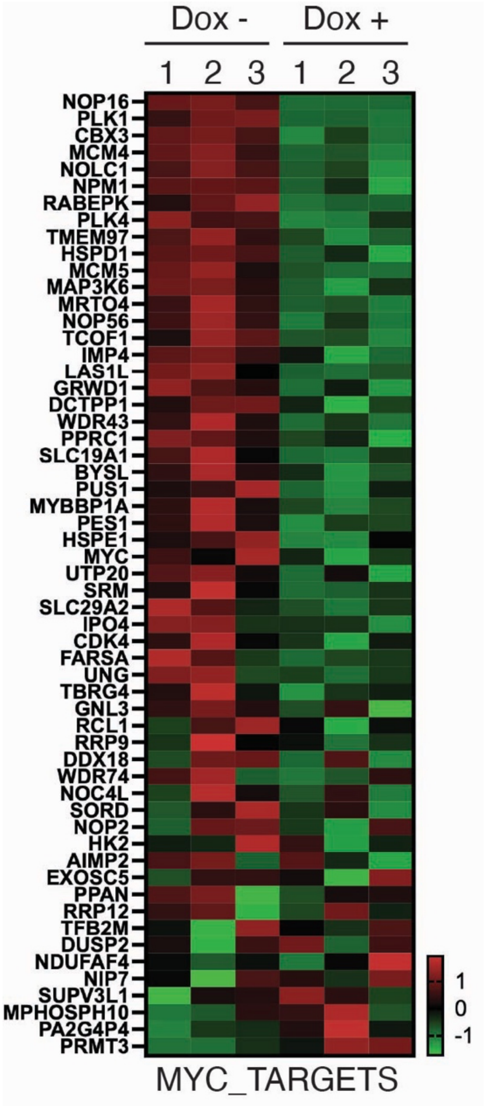

Fig. S1. **Heatmap showing the expression of *c-MYC* signature target genes in response to doxycycline (Dox)-inducible overexpression of ABHD5 in 22Rv1 cells.**
Data correspond to Fig. 1B, with individual gene names labeled as row titles in the heatmap. The *HALLMARK_MYC_TARGETS_V2* gene set from MSigDB was used to define *MYC* target genes.
